# What Should Health Departments Do with HIV Sequence Data?

**DOI:** 10.3390/v12091018

**Published:** 2020-09-12

**Authors:** Ethan Romero-Severson, Arshan Nasir, Thomas Leitner

**Affiliations:** Theoretical Biology and Biophysics, Los Alamos National Laboratory, Los Alamos, NM 87545, USA; eoromero@lanl.gov (E.R.-S.); anasir@lanl.gov (A.N.)

**Keywords:** HIV epidemic, public health, molecular surveillance, phylogenetics, transmission reconstruction

## Abstract

Many countries and US states have mandatory statues that require reporting of HIV clinical data including genetic sequencing results to the public health departments. Because genetic sequencing is a part of routine care for HIV infected persons, health departments have extensive sequence collections spanning years and even decades of the HIV epidemic. How should these data be used (or not) in public health practice? This is a complex, multi-faceted question that weighs personal risks against public health benefit. The answer is neither straightforward nor universal. However, to make that judgement—of how genetic sequence data should be used in describing and combating the HIV epidemic—we need a clear image of what a phylogenetically enhanced HIV surveillance system can do and what benefit it might provide. In this paper, we present a positive case for how up-to-date analysis of HIV sequence databases managed by health departments can provide unique and actionable information of how HIV is spreading in local communities. We discuss this question broadly, with examples from the US, as it is globally relevant for all health authorities that collect HIV genetic data.

## 1. Introduction

An HIV infected person in care in most developed countries will typically have part of the polymerase gene of their HIV population sequenced at least once, and often multiple times, to help guide clinical/therapeutic decisions. For example, many United States (US) state health departments collect HIV sequence data from healthcare providers and laboratories as a matter of state law and then communicate those sequences to the Centers for Disease Control and Prevention (CDC), as part of an integrated HIV prevention and surveillance program [1,2]. In some states, such as Michigan, these collections go back for more than 15 years, containing >10,000 sequences [3]. In California, the Los Angeles County alone received HIV genetic sequences from >20,000 individuals between 2006 and 2016 [4]. Together, the state and county-level sequence collections likely match or possibly outnumber the number of entries in the public HIV sequence database (859,952 as of July 2020) that contains most published HIV sequences. Collectively, these databases likely represent the single largest, most well sampled, document on the historical and statistical properties of the HIV epidemic as it plays out in locations ranging from dispersed rural communities to large urban centers in the US and worldwide. Nevertheless, using these databases for public health surveillance is a complicated issue that needs to balance individual and public benefit with the potential harm to individuals and communities from misuse of these resources [5,6].

In this paper, we address the question, what should public health departments do with HIV sequence data? We begin by stating the potential benefits and risks of using HIV genetic data in public health surveillance and then discuss three reasonable options. We are not arguing for a “one-size-fits-all” approach, but rather, our aim is to delineate both the motivation and benefits of different analysis levels that can make use of these rich collections of HIV genetic data. We discuss this sensitive question primarily in the context of the US HIV epidemic, but the issues, benefits, and risks associated with these analyses are broadly applicable to many other countries that also mandate collection of HIV genetic data from laboratories and clinics.

## 2. Genetic Tracking of HIV

Surveillance is key to prevent the future spread of infectious diseases. This is evident in the case of COVID-19 where a three-pronged approach of test, trace, and isolate has proven successful in containing virus spread in some Asian countries. In the case of HIV, surveillance requires a coordinated effort from healthcare professionals, state and local health departments, community members, and scientists. At the very basic level, healthcare providers notify increased patterns of HIV diagnosis in a geographical region, population, or community to the state and local authorities. This is often followed by contact tracing investigations, as permitted by local resources, to trace the epidemiology of the suspected outbreak and to identify additional at-risk and undiagnosed individuals within the contact network of new patients. Similar efforts focused on getting HIV-positive individuals back into care and virally suppressed may also be enacted. Because the median time-gap between HIV contraction and clinical diagnosis is three years [7], on-field surveillance cannot always identify and respond to HIV outbreaks in real-time. Moreover, patients cannot always accurately recall the source of HIV transmission, especially if they acquired the virus long ago. Traditional surveillance is therefore passive and can miss several undiagnosed HIV-positive persons involved in the outbreak. According to CDC, these individuals may contribute ~40% of new HIV transmissions [7].

**Figure 1:**
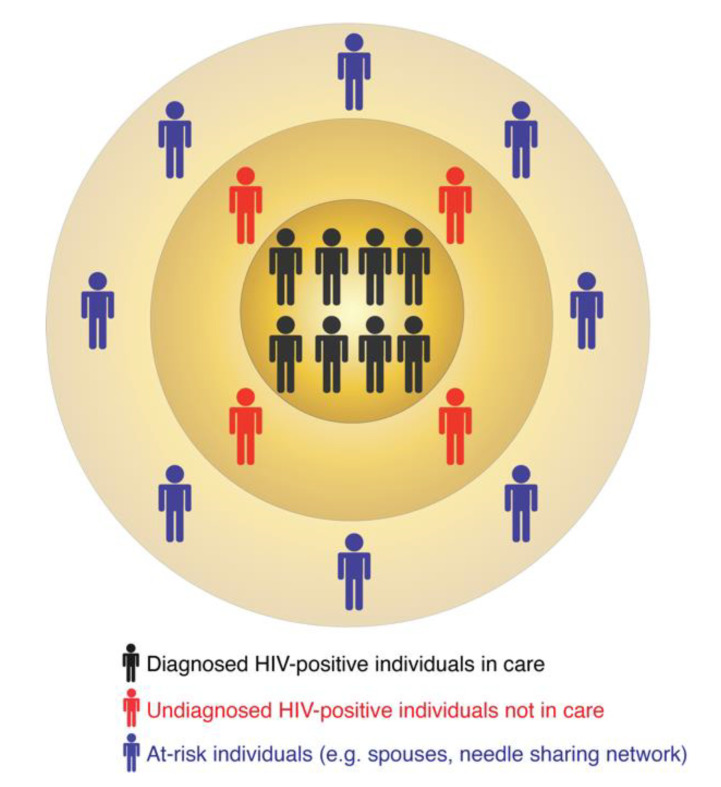
The multi-layered structure of HIV transmission clusters.

In turn, molecular surveillance is an active approach that is now a routine component of HIV prevention strategies implemented by several US states and the CDC. Molecular surveillance leverages information from the HIV genetic data collected at the state and county levels. Because HIV evolves continuously within an infected person [8,9,10], individuals harboring closely-related viral sequences are possibly linked by direct or indirect virus transmission [11,12]. Such groups of linked sequences form molecular or genetic clusters, which may indicate recent and ongoing HIV transmissions [13,14,15,16]. A molecular cluster does not indicate the source of virus transmission, i.e., who infected whom. It simply includes diagnosed HIV-positive individuals with closely-related viruses. The similarity among virus genetic sequences can result from one or multiple virus transmission scenarios. For example, all members of a cluster may have been infected by a common unidentified source or by multiple undiagnosed donors in the transmission chain. These are only two of many possible transmission scenarios that may be compatible with the epidemiology of such a cluster. A molecular cluster is therefore usually part of a larger transmission network, for which one or several individuals are yet to be identified, diagnosed, and enrolled in care (Figure 1). These individuals can be identified via on-field investigations similar to traditional surveillance or their existence can be predicted via computational tools (as described below). Thus, the follow-up procedures (i.e., interviewing infected persons and following up on potential partners) in both traditional and molecular surveillance can be quite similar and may lead to identification of additional HIV-positive or at-risk individuals.

The recent CDC guidelines require that CDC funded jurisdictions run monthly analyses of molecular cluster detection using CDC recommended computational tools [1,12]. The automatization of this process can aid in optimizing resource allocation and direct follow-up investigations to localities where they are most needed. Moreover, molecular surveillance is independent of patient recall history and is thus a faster and less expensive way to rapidly, and hopefully in real-time, detect and stop emerging outbreaks.

## 3. Risks and Concerns of HIV Genetic Tracking

Molecular surveillance is neither exclusive nor new to HIV. Due to recent technological advancements in low-cost, high-throughput genome sequencing technologies, genomic data for large numbers of samples can now be generated timely and at low cost. These genomic datasets provide clues regarding pathogen evolution and spread in human and animal populations and enable healthcare professionals and scientists to forecast and prevent future outbreaks. However, due to the sensitivity and stigma associated with HIV diagnosis, there are real risks associated with genetic tracking of HIV spread in populations [17,18]. It is important to note that the risks associated with genetic tracking of HIV are similar to name-based reporting that is common in all US states. For example, information obtained thorough named-based reporting such as contact tracing and some of the information gained from molecular surveillance may be sensitive information, such as the grouping of individuals linked by virus transmission or that certain groups have a higher burden of HIV transmission than others. While this information is important for public health decisions on prevention efforts, this type of information can be abused in the wrong hands, especially in countries where identification of HIV infected persons or routes of infection can lead to physical, legal, or social threats. For example, an HIV-positive, undocumented person living in the US could be charged with a crime, forming the grounds for deportation, for having sex even if appropriate protections are used [19]. In this and similar environments, identifying the movements of infection in a population is a risk to real people. Other potentially vulnerable and marginalized populations such as men who have sex with men (MSM), sex workers, and transgender persons may fear prosecution and exposure based on mere allegations of intentional virus transmission. The potential harm can also exist on the population level. For example, a recent study suggested that transgender women in Los Angeles County were more likely to be involved in HIV transmission networks [4]. Although the study did not establish that transgender women were more likely to spread HIV, these associations, however wrong or misinterpreted, can elevate the stigma associated with populations that need public health services [20]. The reality in the US is that 35 of 50 states have laws criminalizing the potential of HIV transmission via sexual or non-sexual contacts [19,21] even when the risk of transmission is non-existent [22]. These laws are not simply “on the books” but are actively used to charge and convict people. In this environment, the risk to both individuals and communities is very real and cannot be dismissed lightly [20]. In addition, most patients are likely unaware that their data is being communicated to state and federal agencies and used by parties other than the clinic or laboratory providing care. While public health surveillance does not require informed consent in the same way as medical research [23,24], this practice may be perceived as a violation of patient privacy and can increase public mistrust in the government and medical practice. It may even discourage at-risk individuals to enroll in HIV care and thus cause more harm than good. Similarly, the nature of protocols in place to safeguard patient data and confidentiality are not completely known to the public. Finally, use of genetic sequence data to describe the flow of infection in populations may need to involve more than just an informed population but rather direct involvement of community representatives in the decision-making process that we address in this paper. The imperative of public health action is to protect the public health, but the nature of infectious diseases makes the ethical calculus involved difficult. However, it is not necessarily true that doing nothing is the best option.

Given this central tension between the duty of care to both individuals and populations with the need to reduce the spread of HIV, we introduce three illustrations of the potential use of HIV genetic sequence data in public health practice. These include (1) minimal analysis focusing only on directly measurable quantities (e.g., drug resistance mutations) and methods that describe the (2) statistical and (3) historical dynamics of evolving epidemics (Table 1).

### 3.1. Minimal Analysis

If the risk of harm to individuals and communities is considered to outweigh any potential benefit, then genetic sequence data can still be used, but only in a limited, minimal way. These types of analyses would focus on aggregate properties of a sequence database such as changes in the transmission of HIV-1 subtypes or the prevalence of drug resistance mutations. These kinds of analyses give some sense of population-level health parameters with respect to a clinically relevant outcome (e.g., initial infection with a drug-resistant virus) while avoiding many of the potential risks associated with HIV sequence analysis for public health.

#### 3.1.1. Arguments in Favor of Minimal Analysis

The primary argument for doing minimal analysis of HIV sequence data at a public health department level is that the potential harms to both the community and the individual are greater than the potential benefits of any deeper analysis. The analyses that we discuss below work by attempting to either reconstruct the flow of infection between and within sub-populations or the historical record of who likely infected whom and when. Both of these kinds of information have the potential to cause harm by potentially stigmatizing communities or making individuals susceptible to legal and criminal repercussions.

One advantage of minimal analysis is that it does not require complex models, and the results generally have unambiguous interpretations. That is, any quantity that we can compute from the sequence data directly (i.e., the temporal/spatial dynamics of drug resistance or the rise and fall of specific HIV subtypes) can be interpreted as a description of temporal trends in the surveillance data rather than as a complex statistical inference procedure. The case for minimal analysis also includes arguments against more sophisticated analysis. As we will discuss later in this paper, more sophisticated analyses must include uncertainty from multiple sources such as in the reconstruction of a phylogenetic tree and mapping phylogenetic trees to transmission histories. Moreover, more sophisticated analysis can be based on highly subjective assumptions. That is, if we give one data set to multiple scientists, we may get multiple, possibly contradictory results that cannot be objectively ranked without diving into the technical aspects of the analyses. In fact, this is exactly what happened in a recent multi-team study with simulated HIV sequence data [25]. A final epistemic argument for minimal analysis is that the potentially long delay between infection and diagnosis means that the information from even the most up-to-date analyses will be in some cases years old. While all analyses of HIV data are affected by delays in discovery of new cases, minimal analysis is not intended to actively direct resources to specific outbreaks or to recommend active changes in policy and is therefore less susceptible to delays.

#### 3.1.2. Arguments Against Minimal Analysis

**Figure 2:**
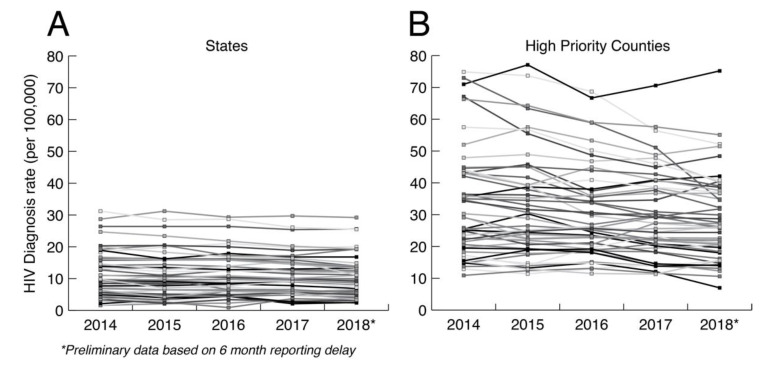
HIV diagnosis rate per 100,000 at the (**A**) state and (**B**) county levels. The county data includes diagnosis rates for 48 counties, Washington, DC, and San Juan, PR, that have been prioritized for first year ending the HIV epidemic. Data taken from CDC (https://www.cdc.gov/nchhstp/atlas/index.htm, 30 January 2020).

The primary argument against minimal analysis is that we are grossly underusing public health HIV sequence collections to understand how HIV is spreading at the local level. Ultimately, HIV prevention is a local issue that requires adaptation to local populations. In the US, HIV transmission is highly heterogenous, including major demographic, geographic, and cultural distinctions such as sexual orientation, population density, race, education, and geography. Figure 2 shows the HIV diagnosis rates at both the state and county levels in the US controlling for the relevant population sizes. HIV diagnosis rates at the county level are much more variable between counties and within counties over time than at the state level. Aggregating data up to the state level hides heterogeneities that matter for local prevention, which are only apparent at smaller scales. Having a greater understanding of how HIV is actually spreading in local populations is necessary for curtailing its spread and for adapting prevention methods to future trends. Thus, HIV prevention needs to be agile, local, and adaptive to changing dynamics.

### 3.2. Transmission Dynamics Analysis

Transmission dynamic analysis focuses on fitting models that describe the broad patterns of virus transmission in a population in terms of mathematical expressions using both genetic sequence and standard epidemiological data such as diagnosis time series. These methods are often referred to as phylodynamic methods [26] because they are generally (although not always) based on first inferring a phylogeny from genetic sequence data and then fitting an epidemiological model to that phylogeny [27]. The logic behind this type of analysis is that a mathematical model can potentially tell us quantities that are highly relevant but difficult to determine directly, e.g., incidence stratified by variables such as race and age and how certain risk groups are mixing with one another. There are a wide range of methods for conducting this type of analysis that make different assumptions and are more or less suitable for addressing epidemiological questions [28,29].

#### 3.2.1. Arguments for Transmission Dynamic Analysis

The simplest argument for transmission dynamic analysis is that there is a large and well-documented body of literature showing that transmission dynamics of HIV and other pathogens can be modeled in terms of the kinds of data that are typically available to a public health department, e.g., partial *pol* sequence data and standard epidemiological data such as case counts. Here, sequence data relates to what is happening in a population at a large, macroscopic level. An early example of this type of analysis was able to estimate the proportion of new HIV cases that were coming from both acutely infected and undiagnosed persons in Detroit over time [3]. Both of these parameters are important for both predicting and monitoring the efficacy of new prevention programs such as Pre-Exposure Prophylaxis (PrEP) and are not readily obtainable from traditional surveillance data.

Transmission dynamic analysis generally minimizes the risk of identifying transmission pairs in the databases. That is, fitting a mathematical model of transmission to genetic sequence data can describe the broad processes of transmission in a population but not necessarily the specific transmission history of a given sample. Formally, a transmission model fit to a set of genetic sequence data could be used to calculate the probability that two persons in the sample infected one another. However, transmission dynamics analyses are generally based on a sub-sample of the data that are unlikely to contain any direct transmission pairs. In practice, transmission dynamic analysis does not allow us to make any claims about who infected whom in a given sample and therefore carries a minimal risk of inadvertently identifying transmission pairs.

Recently, the results of a large-scale simulated data challenge investigating different transmission dynamics analyses of HIV genetic sequence were published [25]. The simulated data included a wide range of different contexts including populations with ongoing HIV prevention interventions. While different methods showed highly variable quality in their results, a single team was able to correctly estimate HIV incidence, change in incidence due to intervention, and proportion of infections coming from recently infected persons in most of the simulated data scenarios.

#### 3.2.2. Arguments against Transmission Dynamic Analysis

**Figure 3:**
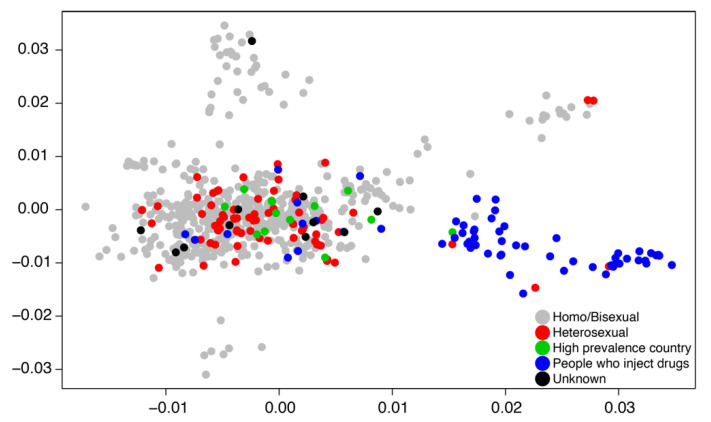
A two-dimensional principal component plot describes clustering patterns of Swedish HIV subtype B cases sampled between 2002 and 2010 [30]. Data points are labeled by risk group.

A basic weakness of transmission dynamic modeling in general for any data including genetic sequence data is that the model applies globally to the domain of the data. In practice, when we are fitting transmission models to genetic sequence data, we subsample the data for both computational and theoretical reasons such that the data represent a broad swath of the population in both geography and time. Focusing on the broad population dynamics may hide essential heterogeneities by aggregating up to a sufficient level for the transmission model to work. Likewise, dividing the population a priori into transmission groups such as MSM and people who inject drugs (PWID) may prevent understanding how sub-epidemics communicate with one another and whether or not there are possible intervention strategies at the boundaries of a priori population divisions. Figure 3 illustrates that HIV transmissions can spillover between PWID and sexual transmission groups.

From a pragmatic perspective, transmission dynamic analysis of HIV genetic sequence data is difficult to implement and requires direct programing of mathematical models and statistical inference methods. There is increasing convergence towards more user friendly and “off the shelf” type uses of transmission dynamic analysis. However, these types of analysis are not trivial to implement and can lead to misleading results if they are not constructed carefully.

### 3.3. Documenting Historical Epidemiology

Rapid progress in the analysis of genetic sequence data has led to an explosion of off-the-shelf methods for describing, in a range of detail, the historical dynamics of evolving epidemics [31,32,33,34,35]. That is, we have methods that allow us to reconstruct how an epidemic has played out in the past based on contemporary and historical genetic sequence data. Assuming fairly constant evolutionary rates, evolutionary theory predicts that sequences separated by a few mutations are more closely related than those separated by many mutations. Thus, the fundamental principle that is exploited here is that HIV rapidly accumulates mutations over time, which results in persons who have recently infected each other harboring HIV that is separated by fewer mutations from each other than from persons who are further away in the transmission chain. Hence, by comparing HIV sequences from persons in a public health database one could extract information about transmission heterogeneity in a population. Tight clusters would indicate recent transmissions among socially connected individuals, while HIV sequences further away might indicate separate transmission communities and distant sources of infection. These analyses therefore attempt to probabilistically infer the transmission tree or, synonymously, the transmission history of a sample, that is, the record of who transmitted to whom and when (see [36] for comparison of existing methods).

#### 3.3.1. Arguments for Documenting Historical Epidemiology

While identification of genetic clusters can find closely related transmissions and thereby guide prevention efforts, evolutionary theory has more to offer. Recently developed phylodynamic methods maximize what we get out from genetic clusters and data [37]. These analyses can identify the underlying transmission network structure, resolve epidemiological links between groups of individuals [11,38], predict risk factors for virus transmission, and estimate the numbers of missing and undiagnosed persons involved in HIV outbreaks [31]. These benefits can significantly enhance HIV surveillance and prevention efforts and yield direct and actionable insights. For example, clustering patterns observed in the same cluster can be consistent with multiple transmission scenarios. All members may have been infected by a single person or by multiple undiagnosed individuals. The latter emphasizes the need to identify undiagnosed HIV-positive persons involved in the outbreak to limit future spread. A cluster that may indicate existence of a large number of undiagnosed persons may demand immediate attention compared to a cluster that is nearly fully sampled. Thus, knowledge of the underlying transmission network structure can guide health agencies to prioritize clusters with the maximum potential of future spread. This knowledge can save many lives by alerting at risk individuals and enrolling undiagnosed HIV-positive individuals into care. Similarly, node labeling of age and gender, dates of diagnosis, and area zip codes on phylogenies can dramatically alter our understanding of transmission risk factors. For example, clusters where old individuals are infecting young MSM may suggest different actions. Recent advancements in phylodynamic methods now allow analysis of partially sampled outbreaks and also account for within-host virus diversity [31,39]. These features therefore significantly enhance the kind of information we get from HIV sequence collections and standard clustering analyses. The analyses can also infer the number and contributions of exogenous virus introductions as a result of migration or international travel in different states and countries. This information can further guide public health response to disease emergence.

#### 3.3.2. Arguments against Documenting Historical Epidemiology

These analyses are computationally demanding and therefore cannot (yet) deal with extremely large datasets. A major argument against the use of historical analyses is that these analyses can potentially identify transmission pairs and thus predict the direction and directness of virus transmission among sampled individuals. As stated above, this knowledge can be extremely important for public health decisions on prevention efforts but can be abused in the wrong hands. It can lead to community distrust, fewer diagnoses, increased stigma, and ultimately more HIV transmissions and fewer people that receive antiviral treatment [6]. In jurisdictions with criminal HIV laws there is also the concern of prosecution of transmitting HIV [21]. While these analyses are inherently uncertain and are based on probabilities, this distinction is easily lost when communicating the results of these kinds of analyses. It is therefore important to clarify that “who infected whom” is a statistical statement about what the model “thinks” and should not be confused with a legal statement. The colloquial interpretation of probabilities as a form of belief may be at odds with the technical use of probability as a measure of uncertainty, which can lead to unintended implications. However, the reconstructing of epidemiological history from pathogen genetic sequence data is *fundamentally* uncertain in that the genetic sequence data can never in practice perfectly constrain a phylogenetic tree that forms the basis of such a reconstruction. That is, a reconstruction of an epidemiological history based on real data that properly considers uncertainty from all sources such as the uncertainty in the underlaying phylogeny will almost never lead to statements such as “we are 95% certain that A infected B”. We illustrate this idea in the next section where we show how a phylogenetic reconstruction might work and the kinds of information that we would get from such an analysis.

## 4. An Image of a Phylogeny-Based HIV Surveillance System

**Figure 4:**
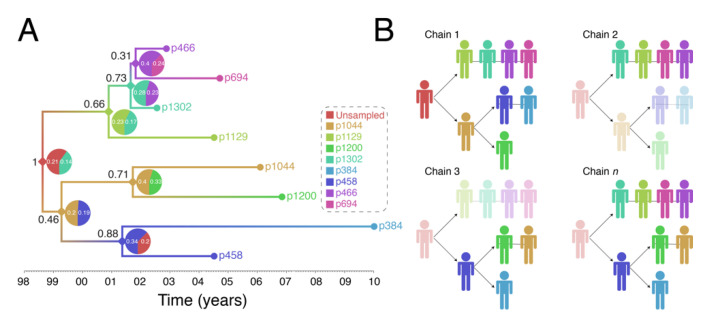
Reconstruction of Swedish HIV transmission chain 79 (c79) involving 8 MSM. (**A**) SCOTTI (ver. 2.4.7) [31] was used to infer the transmission tree. SCOTTI uses statistical and Bayesian frameworks to model host-to-host transmissions as between-host virus migration events, while accounting for within host evolution and undiagnosed hosts. To comply with SCOTTI requirements, an exposure interval was explicitly defined for all individuals. This interval limits virus exposure to the period between when a host was first infected (exposure start date) and last infectious (exposure end date). This information was not available for all individuals. We therefore assumed that individuals stopped being infectious the day they donated a blood sample for *pol* sequencing after testing seropositive for HIV. Exposure begin date was then set to 10 years prior from the exposure end date. The HKY+G model was identified as the best fitting evolutionary model (AIC score = 3194) by the LANL FindModel implementation of ModelTest [41]. The SCOTTI Python script was modified to allow for gamma distributed rate variation across sites. SCOTTI configuration files were run in BEAST (ver. 2.5.2) [42] for 10 million iterations (10,000 trees sampled). A maximum clade credibility (MCC) tree is shown after 10% burnin. (**B**) Multiple virus transmission pathways are possible from the tree in (**A**). A few examples are shown to illustrate the underlying statistical uncertainty in inferring who infected whom.

**Figure 5:**
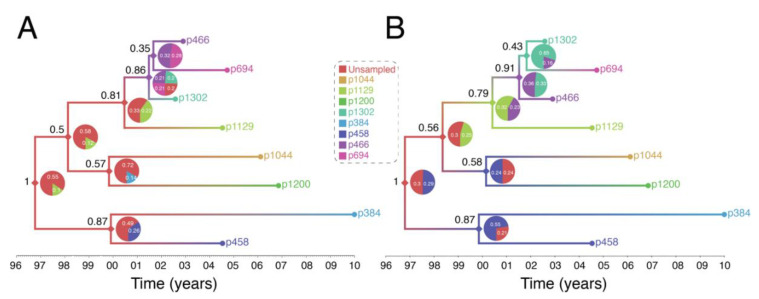
Reconstruction of Swedish HIV transmission chain 79 (c79) [30] using revised assumptions. (**A**) The exposure interval was constrained by the seronegative and seropositive dates for each individual. When the seronegative date was either unknown or was >10 years before the seropositive date, exposure start date was set to 10 years, as in the previous example in Figure 4. (**B**) Exposure start date was set to actual seronegative dates regardless of how many years prior they occurred to the seropositive date. Exposure end date was set to the most recent seropositive date in the dataset. This interval therefore allowed most individuals to transmit the virus even after testing HIV positive and widened the possible exposure intervals for all individuals.

**Figure 6:**
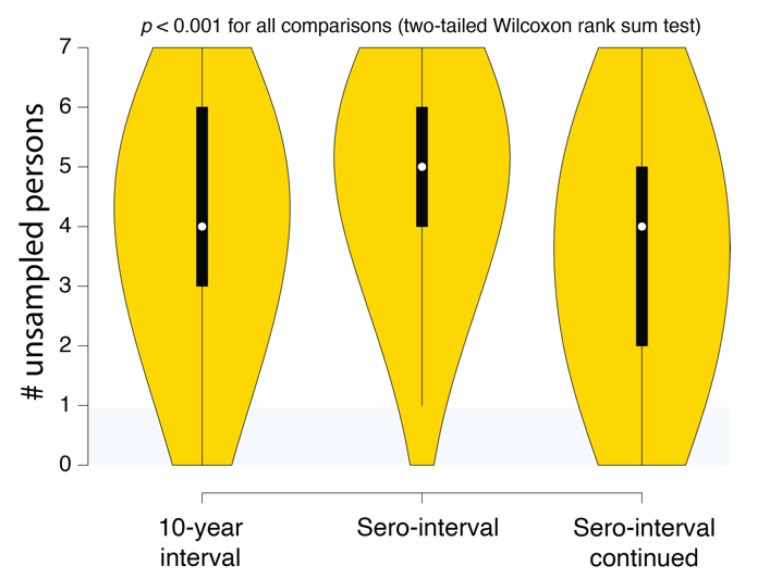
SCOTTI estimates of the number of undiagnosed persons in different Swedish HIV transmission chain 79 (c79) transmission history reconstructions. The intervals correspond to the 10-year exposure interval (Figure 4A), the sero-interval restricted by HIV-positive and negative test dates (Figure 5A), and the open sero-interval to allow individuals to transmit HIV even after testing HIV-positive (Figure 5B).

Figure 4 shows output of a transmission history reconstruction of a previously published HIV transmission cluster [40]. The cluster includes 8 MSM in Stockholm (sampled between 2002 and 2010) that were believed to have infected one another based on relatively close genetic distances between their HIV sequences. Figure 5 shows the reconstructed transmission history under a different set of assumptions and using different levels of data. Figure 6 shows the estimated number of persons that were involved in the transmission cluster.

We want to make 4 specific points with these illustrations: (1) reconstructed transmission histories provide an image into the otherwise hidden transmission story of a genetic cluster, (2) the reconstructed transmission histories have a substantial-level of uncertainty about exactly who infected whom, (3) reconstructed transmission histories are sensitive to both methodological assumptions and the available data, and (4) reconstructed transmission histories provide actionable information that can be used to both make public health decisions and better understand the local HIV epidemic.

*(1) Reconstructed transmission histories provide an image into the otherwise hidden transmission story of a genetic cluster.* The transmission tree in Figure 4A represents the best guess of who transmitted to whom when based on the available data including HIV genetic sequences. Infected persons are indicated by a unique color and an anonymous patient ID. Each internal node in the tree represents a transmission event occurring between two people at a certain time. The color at an internal node indicates the most probable infector for the given data and methodological assumptions. Put into words, the transmission history in Figure 4A documents how the 8 sampled persons became infected. Under the most likely arrangement given the data, in 1999, an unsampled person infected p1044 who, about 2 years later infected both p458 and p1200. p458 then infects p384 in 2001 who is about 20 at that time. The unsampled case that started this outbreak also infects p1129 around the same time he infected p1044 (2001). P1129 then begins a chain of transmission leading to p1302, then to p466, and finally p694. Interestingly, both p1129 and p458 were the oldest persons in the dataset (data not shown). This tree tells us that not only are these 8 persons “linked” but exactly who and approximately when each one was infected, and, furthermore, that the most probable inference is that at least one person is missing from this cluster.

*(2) The reconstructed transmission histories have a substantial-level of uncertainty about exactly who infected whom.* The above narrative is only the most probable reconstruction. In fact, even under this fixed set of assumptions, there is substantial uncertainty in who, exactly, is infecting whom. The pie charts at each node indicate the uncertainty in who is the donor for that transmission event. While multiple infectors are possible, only two most probable are illustrated for clarity. The numbers indicate the number of times that we sample a transmission history where one of the persons is the donor. Likewise, the number in black next to each node is the number of times that node itself is sampled. For example, the node connecting p466 and p694 in Figure 4A only shows up in the analysis about 31% of the time and of those 31% of the time, the donor is inferred to be p466 about 40% of the time. While the data suggest that p466 is the most likely infector of p694, we only sample this particular transmission about 31% * 40% = 12% of the time. Figure 4B illustrates multiple transmission chains compatible with the tree in panel A. In general, when we reconstruct transmission histories from genetic sequence data, we can make a case for what is most plausible given the data, but we also can compute that the probability of exactly that sequence of events occurring is low. This is due to the fact that for even a small number of linked cases, there is an astronomically high number of ways that they could have infected one another especially when you consider that unsampled persons may also be involved. Genetic sequence data constrain the possibilities but cannot elevate one possible outcome to the level of certainty. While we are neither lawyers nor legal scholars, we do not believe that these kinds of analyses even with nearly perfect data rise to the level of being legal evidence of direct transmission. However, even given high levels of global uncertainty, we can discern common features of transmission in such clusters; for example, we may observe a trend whereby older men are transmitting HIV to younger men.

*(3) Reconstructed transmission histories are sensitive to both methodological assumptions and the available data.* This issue is expanded by considering that different kinds of data and assumptions can go into the analysis. Figure 5 illustrates this idea. In Figure 4A, we assume that all we know is the genetic sequence data, and we make the additional assumption that each sampled person was infectious for no more than 10 years prior to testing positive, which is reasonable on average for HIV. In Figure 5A, we re-run the analysis but restrict the potential window that each person is potentially contagious to include previous negative HIV tests, which are known for 5 of the 8 individuals. Finally, in Figure 5B we relax the assumption that people do not transmit after they are diagnosed. The effects of including more data (i.e., previous negative HIV tests) and relaxing the assumption no transmission occurs after diagnosis changes our reconstruction of the transmission history. This occurs in part because the additional data further excludes certain possibilities, changing our reconstruction of the transmission history. For example, Figure 5A now suggests that one or more unsampled persons started the two chains of HIV transmission we described above. Taking the inherent uncertainty in these analyses together with sensitivity to subjective assumptions, we believe that these kinds of analyses cannot be used to establish by any standard that a given person must have infected another person. Such strong statements are simply outside of the domain of this kind of work.

*(4) Reconstructed transmission histories provide actionable information.* While uncertainty is an inherent part of epidemiological reconstruction, reconstruction yields not only insight but actionable results that are not obtainable from other kinds of analysis. An example of this kind of result is illustrated in Figure 6. This plot shows the distribution of the number of missing cases in this transmission cluster. Being able to quantify the evidence for missing cases allows public health agencies an ability to figure out not only how many undiagnosed persons might exist in the population but also where to look for them. Accounting for these unsampled persons can prevent further spread of HIV in the community. For example, if we reconstructed the history of multiple putative transmission clusters, we could prioritize limited contact tracing resources to cases that are likely to have contacted an undiagnosed person. Likewise, these reconstructions can reveal macroscopic patterns or population behaviors that can better guide public health response to HIV epidemics.

We believe the answer to the motivating question behind this paper—what should health departments do with HIV sequence data—may be complex and, critically, that the deliberation needs to be based on a solid understanding of both the utility and the limits of the analytical methods involved.

## 5. Ethical Aspects of Genetic Tracking of HIV Transmissions

We have described three different strategies that can use HIV genetic data collected by US states, counties, and other countries, to help contain HIV spread. These options are summarized in Table 1 and provide varying levels of actionable information. We believe that the maximum and most actionable information is provided by phylogenetically enhanced HIV surveillance systems that attempt to reconstruct the virus transmission history among clustered individuals as we have illustrated in an example analysis of a small HIV transmission cluster. These kinds of analyses can provide immediate actionable information to contain future virus spread and also enroll undiagnosed individuals into healthcare. However, linking of cases by virus transmission is a sensitive issue, and it is important to communicate the value of these insights to both public health practitioners but also to the communities whose data are being used and preferably include them in the decision process [5,6].

One possible approach to minimizing risk to individuals and communities would be to only record and use information that does not involve any statements (even probabilistic) about who transmitted to whom. For example, Figure 6 illustrates the use of how reconstructing epidemiological history gives an estimate of the number of undiagnosed persons that belong to that cluster but are not in the public health database. Using this information to triage contact tracing resources would not involve making any specific claims about who infected whom. Likewise, individual transmission clusters could be ranked on the levels of superspreading without having to rely on a single reconstructed history [43]. It is important to understand the probabilistic nature of these inferences, but if they are deemed to be too risky, there are still methods for obtaining useful information that uses epidemiological history reconstruction methods.

Information derived from phylogenetic analyses should be available to persons with a need to know. Hence, it is similar to medical records, held securely and only available to relevant medical staff. Phylogenetic information about genetic relatedness among HIV from different patients is similar to the statements about “who might have infected you” that partner services record as part of contact tracing. This kind of information could be stored using similar protocols for existing sensitive data.

Finally, public health staff and scientists should inform the public about their efforts to improve disease prevention. A close collaboration between community interests and public health interests promotes efficient prevention of further HIV spread.

## 6. Conclusions and Recommendations

Here, we show that there are public health benefits from applying evolutionary theory principles to allocating prevention resources and fighting the HIV epidemic. Because linking transmissions among infected persons is sensitive information, we also discuss possible public health risks associated with such information. Preventing further spread of HIV is in the interest of the society and welfare of the people, yet doing so must not cause excess harm. Disease prevention is a complex and difficult task, with limited resources and many unknown factors. Modern evolutionary theory can be used to better trace and predict HIV epidemics, identify local and changing risk factors, and thereby direct allocation of public health resources to where they are needed most. Inference based on HIV genetic sequences provides useful epidemiological information that is actionable for public health purposes. It is sensitive information about potential transmission linkages between individuals and groups. It is important that such information is securely stored and not misused.

## Figures and Tables

**Table 1:** Summary of three different options to analyze state- and county-level HIV genetic data.

Options	Benefits	Limitations
Minimal analysis	Less complex Simple interpretation Minimizes use of individual data	Underuse of resources Limited actionable information Limited resolution
Transmission dynamic analysis	Macroscopic view of HIV transmission in sub-populations Little or no risk of identifying transmission pairs	Limited understanding of how sub-epidemics may communicate with each other Less applicable to individual transmission clusters Limited “ready to use” software options
Documenting Historical Epidemiology	Maximum use of local HIV sequence collections Identify missing or undiagnosed persons Identify transmission risk factors Yield actionable insights	More complex, less certain Can identify direction or directness of transmission Misuse can discourage enrollment into HIV care and stigmatize populations May require computing resources beyond basic desktop computers
